# Upon the Diagnosis and Treatment of Croup

**Published:** 1854-03

**Authors:** 


Upon the Diagnosis and Treatment of Croup.—By Dr. Hirtz.—Before the labors of modern physicians had assigned to croup its true anatomical characteristics and nosological rank; when, yet confounded among the numerous family of laryngites, it formed a part of their history, it would have been very easy, and consequently quite uninteresting, to furnish cases of cure. Every physician then counted his fortunate cases by the dozen, and every family could cite cases of children attacked by croup and cured at least ten times.

Now, on the contrary, that the name of the true croup is only applicable to pseudo-membranous laryngitis, and that the progress of science has invested its diagnosis with such precision that it is impossible for an experienced physician to mistake it from the very commencement, croup has become a comparatively rare disease, and its cure an exception occurring at long intervals.

On the other hand, the scientific views of the progress and diagnosis of true croup are not yet sufficiently vulgarized among physicians as to render confusion impossible. It still to often happens, that it is mistaken in its insidious approach, and lulls the family

---

and physician into a false security, and then shows itself with all its terrors in a few hours afterwards. In other cases, stridulaus laryngitis is taken for true croup, and draws down upon the unhappy child the whole superfluous arsenal of therapeutics, and excites the unnecessary fears of parents.

Dr. Hirtz, after these just reflections, reports two cases of pseudomembranous croup in two sisters, which terminated in recovery. We shall reproduce only the last.

Case.—Marie R. ——, aged 4 years, of good constitution but temporarily enfeeble by bilious diarrhœa, has been unwell for several days. Every morning she feels fatigued, complains of pain in deglutition ; she is often hoarse but does not cough. The 25th, (the same day on which her younger sister was suffering from croup at its greatest intensity,) Marie appeared more unwell ; she was dispirited and prostrated, more hoarse, and feverish. Dr. Hirtz was seized with a sinister presentiment. Notwithstanding the cries of the child, he examined the throat, and discovered that the soft palate, uvula, and pharynx were red and inflamed, and that the tonsils were already covered by a yellowish-white pseudo-membranous exsudation, which was quite thick, and occupied almost the whole of the surface of each tonsil. There were present, in fact, all the signs of croupal angina. Dr. Hirtz immediately prepared to perform cauterization, and effected this operation completely and satisfactorily in spite of the child's obstinate resistence.

The following night was tranquil; no unfavorable symptom supervened, and on the next morning, Dr. Hirtz hoped that the disease was aborted. This hope was realized ; an abundant mucous secretion was observed in the fauces, deglutition became easy, and the pseudo-membranes, though they continued visible for two days, finally disappeared in copious discharges of mucous. The diptheritis was jugulated.

This case proves how false and dangerous are the ideas which still prevail in society, and even among some physicians, in regard to the incipient symptoms of croup. It is stridulous angina that awakens all the apprehensions of parents ; it is false croup which calls forth all the solicitude of physicians ; it is against this that every mother keeps that precious bottle of hive-syrup which never leaves her.

If there happens to be membranous croup, true croup, on the contrary, there is every chance that the physician is not called until the second or third day, that is to say, when grave symptoms announce that the disease has extended to the larynx.

In these two cases, it required, in the first, not only all the vigilance, but all the forsight of the maternal heart to awake suspicion, and in the second, the existence of croup in the family to cause that disease to be thought of while it was yet time. It will be noticed that the children were running about and playing when the pseudo-membrane was already formed at the entrance of the pharynx.

We cannot therefore too often caution mothers against that dangerous error, which, by mistaking false croup for true croup, causes children to be tormented by violent and superfluous physic, and, which is worse, leads to deception in regard to one of the cruelest and most insidious of diseases.

It is desirable, I say, that it should be generally known, that when a child in full health is suddenly seized, during sleep, with cough and hoarseness, with oppressed breathing and rattling, there is no need to be frightened and to give powerful medicine; for this is not true croup. That if, on the contrary, after a febrile attack, a child has a cold in the head; sore-throat, difficulty of deglutition, and slight hoarseness, the fauces should be examined, or, better still, a physician summoned at once, for nineteen times in twenty true croup commences in this way.

I say nineteen times in twenty, for it is not to be forgotten that croup sometimes commences in the larynx and even in the bronchi, appearing afterwards in the pharynx. I reported a case of this sort twelve years ago; M. Nonat has seen another, and M. Barth a third. But it was on account of their rarity that these facts were published.

When croup attacks one child of a family, the remaining children should be watched with redoubled vigilance; for, whether it be contagious or endemic, the fact is certain that the disease often attacks many members of the same family.

A few words on the treatment of croup. The distinguished physicians who, at the commencement of this century, threw so much light on the nature of croup, attended too exclusively, perhaps, to the mechanical action of the false membrane as a cause of asphyxia. Hence the paramount indication of expelling this foreign body at all hazards, and consequently the use of emetic, which word has become almost the correlative of the word croup. In society especially, this mode of treatment has assumed an exclusive character, and persons believe themselves sufficiently protected if the arsenal of domestic medicines contains tartar emetic.

This doctrine applied in an absolute manner, is false in theory, and dangerous in practice; it is false in theory, because asphyxia may result from many causes besides obstruction of the glottis; it may be explained either by the extension of inflammation to the submucous and muscular tissues of the larynx, producing paralysis of the obturator muscles of the glottis, or by the propagation of diptheritis quite into the bronchi. This is why we often see children die after the expulsion of false membrane, and others recover without this being eliminated. This is why we often observe at the autopsy of children who have died in the last period of croupal asphyxia, that the laryngeal office is sufficiently open for the purposes of respiration. And lastly, this is why tracheotomy is so often unavailing. This doctrine is dangerous in practice, because the reiterated and inopportune employment of emetics is not only useless, but often productive of dangerous disorders.

How can tartar emetic remove a membrane which is scarcely formed and adheres firmly to a surface upon which all moral secretions is checked by a specific inflammation? If it is persevered in, the efforts caused by it increase the cerebral and pulmonary congestion; the stomach is finally worn out and no longer responds to the remedy. The physician returns to the charge with sulphates of copper or zinc, and the child sometimes dies, as Dr. Boeekel has observed, from the intoxication produced by these poisons, rather than by croupal asphyxia. Tartarized antimony given judiciously, however, is a grand resource; but it must be employed with method, and, above all, opportunely, the favorable moment being calmly awaited. This moment occurs when the specific inflammation, modified or checked by other remedies, again permits some secretion of mucus, which separates and detaches the false membranes.

Dr. Hirtz believes that the most rational method of treatment consists in modifying in the first place, the specific inflammation by cauterization; and in combatting it by calomel combined with a few leaches. When this result is obtained, we should act on the mucous secretion by kermes mineral and the golden sulphuret of antimony, and lastly, upon the expectoration by two agents which then become invaluable; the first is tartar emetic, administered discrimination and at proper intervals, and the second is muriate of ammonia (formula of Richter), a true incisive, according to the old momenclature, and a powerful expectorant when administered perseveringly.—*Revue Médico-Chururgicale de M. Malgaigne*, from *Gaz. Méd. de Strasbourg.*

